# Chest pain triage: gut feeling or protocol-based care?

**DOI:** 10.1007/s12471-021-01589-0

**Published:** 2021-05-10

**Authors:** R. T. A. Willemsen, A. W. J. van ’t Hof

**Affiliations:** 1 Department of Family Medicine, Care and Public Health Research Institute, Maastricht University, Maastricht, The Netherlands; 2 Maastricht Heart+Vascular Center, Maastricht University Medical Center+, Maastricht, The Netherlands; 3 Cardiovascular Research Institute Maastricht, Maastricht University, Maastricht, The Netherlands; 4 Department of Cardiology, Zuyderland Medical Center, Heerlen, The Netherlands

With interest, we read the paper by Harskamp et al. in this issue of the *Netherlands Heart Journal* [1]. The authors anticipate enhanced triage of patients presenting with chest pain in primary care in the future. Presently, general practitioners (GPs) perform triage by estimating the urgency based on history taking and physical examination only. They have found an effective and acceptable way of not missing too many major adverse cardiac events (MACEs) by allowing a moderately high referral rate, without compromising the task of effective triage [2].

In primary care, advanced diagnostic means are not available. Earlier attempts to construct clinical decision rules failed because of an unacceptable rise in the number of either missed cases of MACE (reduced safety) or false-positive referrals (increased societal costs and patient anxiety) as compared with the GP’s clinical judgement. In an ambulance setting, the HEART score—which includes troponin measurement—is beginning to stand out as a useful pre-hospital triage tool [3]. Its use in primary care, however, is yet to be determined.

In their paper, the authors describe a population of 664 patients presenting with chest pain during out of hours in a large primary care facility [1]. Retrospectively, two variants of the HEART score without troponin measurement were calculated. In the simplified HEART score, the troponin assessment was omitted, whereas in the HEART-GP score, the troponin assessment was replaced by the GP’s gut feeling (expressed as a low, moderate or high ‘sense of alarm’). This latter score *de facto* increases the weight of the GP’s clinical judgment, because it is included in the ‘History’ element of the HEART score as well as in the added ‘sense of alarm’ item.

Although the HEART score was not studied prospectively and some assumptions had to be made—for example, assuming absence of symptoms that were not mentioned in the patient file—the authors did their best to retrospectively estimate the HEART scores. Altogether, 32 patients (4.8%) had a MACE, either directly upon presentation or at a later time during 6 weeks of follow-up. A MACE rate of 4.8% is in agreement with the results of similar primary care studies. However, a limitation of the study by Harskamp et al. is the exclusion of serious adverse non-cardiac or non-MACE events. The authors reported an additional 18 cases of serious adverse events (pulmonary embolism, heart failure or aortic dissection). It is not clear whether these patients were all included in the referred group.

The authors found a referral rate of 23.6% when GPs performed care as usual (unaided triage), which increased to a virtual 50.0% upon usage of the simplified HEART score [1]. When the HEART-GP score was used with a cut-off value of 3 or 4, the referral rate was 44.1% and 28.0%, respectively. The number of missed cases of MACE was reduced by 83% (simplified HEART score), 83% (HEART-GP score with cut-off value 3) or 50% (HEART-GP score with cut-off value 4), representing a considerable safety gain if these findings are representative of all chest pain patients in primary care.

On the other hand, the absolute effect of referring more patients without an underlying life-threatening disease should not be underestimated. For example, in an earlier study, it was calculated that 860,000 consultations for chest pain are performed annually in primary care in the Netherlands (approximately 17,000,000 inhabitants) [2]. Of these patients, 122,500 are referred immediately, but in 3000 cases (0.3%), a life-threatening diagnosis is initially missed by the GP. Although these numbers include day-time care, with lower referral rates than described in the current paper—which focused on out-of-hours care—calculations based on these data can serve to estimate the effect of using decision aids on patient flow. A reduction of the number of missed cases of MACE by 83% following the usage of the HEART-GP score with a cut-off value of 3, translates into a reduction of 2490 cases of MACE with delayed recognition. However, this comes with a price of almost doubling the referral rate, which would lead to an additional referral of over 100,000 ‘false-positive’ patients in the Netherlands every year. Using the HEART-GP score with a cut-off level of 4 would reduce the number of false-negative cases by 1500, but at the cost of 34,300 patients without a life-threatening disease who are referred annually.

A major limitation of the study by Harskamp et al. is its retrospective design. Patients were categorised in the HEART score groups based on electronic health record data only. In daily practice, it is not the HEART score that decides to refer patients, but it is the GPs, and the HEART score might help them in their decision-making. In the near future, the HEART-GP score should therefore be studied prospectively as an additional tool in decision-making for the full spectrum of chest pain complaints in primary care.

Nevertheless, the current results are promising because of two facts. First, the authors have offered an insight into the possible effect of HEART score–aided triage: a significant reduction of the missed MACE rate without inducing a massive rise in unnecessary referrals. Second, future options to work with troponin assessments in primary care are ‘the remaining rabbit in the hat’ to strengthen the HEART score in primary care, provided that high-sensitive, easy-to-use, portable troponin assays variants become available.
